# Dose- and Time-Dependent Association of Smoking and Its Cessation with Glycemic Control and Insulin Resistance in Male Patients with Type 2 Diabetes Mellitus: The Fukuoka Diabetes Registry

**DOI:** 10.1371/journal.pone.0122023

**Published:** 2015-03-30

**Authors:** Toshiaki Ohkuma, Masanori Iwase, Hiroki Fujii, Shinako Kaizu, Hitoshi Ide, Tamaki Jodai, Yohei Kikuchi, Yasuhiro Idewaki, Yoichiro Hirakawa, Udai Nakamura, Takanari Kitazono

**Affiliations:** 1 Department of Medicine and Clinical Science, Graduate School of Medical Sciences, Kyushu University, Fukuoka, Japan; 2 Center for Cohort Studies, Graduate School of Medical Sciences, Kyushu University, Fukuoka, Japan; 3 Diabetes Center, Hakujyuji Hospital, Fukuoka, Japan; 4 Department of Environmental Medicine, Graduate School of Medical Sciences, Kyushu University, Fukuoka, Japan; 5 Division of General Internal Medicine, School of Oral Health Science, Kyushu Dental University, Kitakyushu, Japan; University of Leipzig, GERMANY

## Abstract

**Objective:**

Cigarette smoking is an important modifiable risk factor for cardiovascular diseases. However, the effect of smoking and its cessation on glycemic control in diabetic patients has not been fully examined yet. The aim of the present study was to examine the association of smoking status with glycemic level and markers of insulin resistance and secretion in patients with type 2 diabetes mellitus.

**Research Design and Methods:**

A total of 2,490 Japanese male patients with type 2 diabetes mellitus aged ≥20 years were divided according to smoking status, amount of cigarettes smoked and years since quitting. The associations with glycemic level and markers of insulin resistance and secretion were examined cross-sectionally.

**Results:**

HbA_1c_ levels increased progressively with increases in both number of cigarettes per day and pack-years of cigarette smoking compared with never smokers (P for trend = 0.001 and <0.001, respectively), whereas fasting plasma glucose did not. On the other hand, HbA_1c_, but not fasting plasma glucose, decreased linearly with increase in years after smoking cessation (P for trend <0.001). These graded relationships persisted significantly after controlling for the confounders, including total energy intake, current drinking, regular exercise, depressive symptoms, and BMI. In addition, a homeostasis model assessment of insulin resistance and high-sensitivity C-reactive protein also showed similar trends.

**Conclusions:**

Smoking and its cessation showed dose- and time-dependent relationship with glycemic control and insulin resistance in patients with type 2 diabetes mellitus. These findings may highlight the importance of smoking cessation in the clinical management of diabetes mellitus.

## Introduction

The association between cigarette smoking and an increased risk of many diseases, including cardiovascular diseases, has been demonstrated [1,2] and is therefore recognized as an inevitably modifiable risk factor. Although the smoking rate in Japanese men has decreased [3], it is still high compared with that of other developed countries [4]. The type 2 diabetes mellitus epidemic and its complications are also a major public health concern in developing, as well as developed, countries [5], especially in Asian countries where smoking is more popular than in western countries [4]. With regard to the relation between smoking and diabetes mellitus, active smoking has been shown to be associated with increased risk of type 2 diabetes mellitus in a dose-dependent manner [6,7]. Increased risk of diabetes mellitus was also observed for former smokers [6,7], whereas the risk was decreased as the time since quitting increased [7]. Regarding patients with type 2 diabetes mellitus, the association of current smoking habits with hemoglobin A_1c_ (HbA_1c_) showed inconsistent results of positive [8,9] and null relationship [10,11]; however, few studies considered the confounding of other important lifestyle factors, such as diet, exercise, and alcohol drinking. Furthermore, the relation between smoking cessation and glycemic control has not been investigated, at least, on an epidemiological basis, although a quite few intervention studies on smoking cessation have been reported [12,13]. Considering the deleterious effect of smoking on diabetes mellitus, it may be beneficial to clarify the relation of smoking with glycemia in diabetic patients for prevention of diabetic complications. In this context, the aim of the present study was to examine the association between smoking status or its cessation and glycemic control in Japanese patients with type 2 diabetes mellitus.

## Methods

### Study participants

The Fukuoka Diabetes Registry is a multicenter prospective study designed to investigate the influence of the modern treatments on the prognoses of patients with diabetes mellitus regularly attending teaching hospitals certified by the Japan Diabetes Society or certified diabetes clinics in Fukuoka Prefecture, Japan (UMIN Clinical Trial Registry 000002627) [14]. A total of 5,131 patients with diabetes mellitus aged 20 years or older were registered between April 2008 and October 2010. The exclusion criteria of the registry were the following: 1) patients with drug-induced diabetes mellitus or undergoing steroid treatment; 2) patients under renal replacement therapy; 3) patients with serious diseases other than diabetes mellitus, such as advanced malignancies or decompensated liver cirrhosis; and 4) patients unable to visit diabetologists regularly. After excluding 261 subjects with type 1 diabetes mellitus, 2095 female subjects, four subjects for whom smoking duration was unavailable, and 281 who had already eaten breakfast, the remaining 2,490 subjects were enrolled in this cross-sectional study. This study was conducted with the approval of the Kyushu University Institutional Review Board, and written informed consent was obtained from all of the participants.

### Clinical evaluation and laboratory measurements

All the measures were taken at baseline assessment. Participants completed a self-administered questionnaire covering their smoking habits, duration of diabetes mellitus, diet, alcohol intake, physical activity level, and depressive symptoms. Based on smoking status, patients were classified as never smokers, past smokers, or current smokers. Never smokers were defined as those who had never smoked. Past smokers were defined as those who smoked before but did not smoke at the time of study registration. Current smokers were further subdivided by number of cigarettes per day (<20, 20–29, or ≥30 cigarettes per day) and pack-years of cigarette smoking (calculated by number of cigarettes per day divided by 20, multiplied by number of years smoked, <30, 30–49, or ≥50 pack-years). Past smokers were subclassified according to the years since quitting (<10, 10–19, or ≥20 years). The dietary survey was conducted using a brief-type self-administered diet history questionnaire regarding the food frequency of 58 items (BDHQ; Gender Medical Research Inc., Tokyo, Japan). The validity of ranking the energy-adjusted intakes of many nutrients has been previously studied in an adult Japanese population [15]. Alcohol intake was classified as either current use or not. Subjects engaging in sports regularly during their leisure time were defined as the regular exercise group. The presence of depressive symptoms was assessed using the Center for Epidemiologic Studies Depression Scale [16], and subjects who scored 16 or more out of 60 points were defined as having depressive symptoms. The subjects were categorized as taking oral hypoglycemic agents, insulin therapy, or none. Blood was collected by venipuncture after an overnight fast. HbA_1c_ was determined using HPLC (Tosoh Corp., Tokyo, Japan), plasma glucose by the glucose-oxidase method, and serum C-peptide by chemiluminescent immunoassays (Kyowa Medex, Tokyo, Japan). High-sensitivity C-reactive protein (HS-CRP) and serum adiponectin were determined by latex immunonephelometry (Siemens Healthcare Diagnostics, Tokyo, Japan; Mitsubishi Chemical Medience, Tokyo, Japan) in 2,139 and 2,489 patients with samples, respectively. Body mass index (BMI) was calculated from height and weight. Waist circumference at the umbilical level was measured in the standing position. β-cell function and insulin resistance were estimated based on fasting glucose and C-peptide concentrations using the HOMA Calculator [17], and expressed as the homeostasis model assessment of β-cell function (HOMA2-%B) and homeostasis model assessment of insulin resistance (HOMA2-IR) in 2,180 patients after excluding 306 patients those with unacceptable levels of plasma glucose (<3 mmol/l or >25 mmol/l) or C-peptide (<0.2 nmol/l or >3.5 nmol/l) [17].

### Statistical analysis

Differences in the mean values or proportions of the characteristics of the studied subjects were tested by unpaired t-test, analysis of variance or chi-square test, as appropriate. The age-adjusted mean values for HbA_1c_, HOMA2-%B, HOMA2-IR, HS-CRP, and adiponectin were calculated by an analysis of covariance. HOMA2-%B, HOMA2-IR, HS-CRP, and adiponectin were log-transformed for the statistical analyses due to having a skewed distribution, back-transformed and presented with their 95% confidence intervals (CIs). The multivariate-adjusted partial regression coefficients and their 95% CIs were determined using a multiple regression analysis, and examined for linear trends using a multiple regression analysis. In the multivariate-adjusted analysis, we included possible confounding factors, namely, age, duration of diabetes, total energy intake, current drinking habits, regular exercise habits, depressive symptoms, oral hypoglycemic agents use, insulin use, and BMI. All analyses were performed using the SAS software package version 9.3 (SAS Institute Inc., Cary, NC). Values of P <0.05 were considered to be statistically significant in all analyses.

## Results

The clinical characteristics of the study participants are presented in Table 1 according to smoking status. Current smokers were younger and had shorter duration of diabetes mellitus compared with never smokers and past smokers. Duration of smoking was longer, whilst the amounts of cigarette smoked (number of cigarettes per day and pack-years of cigarettes smoking) were lower in current smokers than those in past smokers. Current smokers were more likely to have alcohol drinking habits, depressive symptoms, and to be on insulin therapy and were less likely to engage in regular exercise. Both patients with past and current smoking habits tended to have higher waist circumference levels compared with never smokers.

**Table 1:** Clinical characteristics of the study subjects according to smoking status.

	Never smoker	Past smoker	Current smoker	*P* value
N	505	1306	679	
Age (years)	65.6 (10.7)	66.9 (9.2)	61.2 (10.0)	<0.001
Duration of diabetes mellitus (years)	17.1 (10.8)	17.2 (11.1)	14.5 (10.2)	<0.001
Duration of smoking (years)		29.7 (13.8)	41.6 (10.1)	<0.001
Years since quitting (years)		17.6 (13.4)		
Number of cigarettes per day		31.0 (21.3)	21.4 (11.9)	<0.001
Pack-years of cigarette smoking		47.5 (39.4)	43.8 (26.1)	0.03
Total energy intake (kcal/day)	1835 (477)	1830 (507)	1805 (519)	0.48
Current drinker (%)	49	57	60	0.001
Regular exercise (%)	73	78	64	<0.001
Depressive symptoms (%)	7	6	10	0.006
Oral hypoglycemic agent use (%)	65	63	62	0.75
Insulin use (%)	22	26	30	0.01
BMI (kg/m^2^)	23.4 (3.4)	23.6 (3.0)	23.8 (3.6)	0.13
Waist circumference (cm)	84.7 (9.0)	85.8 (8.3)	86.4 (9.7)	0.004

Abbreviations: BMI, body mass index.

The values are expressed as the means (SD) or percentages.

Table 2 shows the age-adjusted mean values and partial regression coefficients (95% CIs) of fasting plasma glucose (FPG) and HbA_1c_ according to smoking status. The FPG levels did not differ significantly among the groups, whereas HbA_1c_ levels were higher in current smokers, with a mean increase in the current smokers relative to that in the never smokers of 0.20%. Significant elevation in HbA_1c_ levels persisted after controlling for the confounding factors, namely, age, duration of diabetes mellitus, total energy intake, current drinking habits, regular exercise habits, depressive symptoms, use of oral hypoglycemic agents, use of insulin, and BMI.

**Table 2:** Age-adjusted mean values and multivariate-adjusted partial regression coefficients (95% CIs) of FPG and HbA_1c_ according to smoking status.

	Never smoker	Past smoker	Current smoker
FPG (mmol/l)	7.72 (0.09)	7.69 (0.06)	7.84 (0.08)
β (Age-adjusted)	0	−0.03	0.11
95% CI	Referent	−0.25, 0.18	−0.13, 0.36
β (Multivariate-adjusted)	0	−0.08	0.03
95% CI	Referent	−0.29, 0.13	−0.21, 0.27
HbA_1c_ (%)	7.27 (0.04)	7.28 (0.03)	7.47 (0.04)
β (Age-adjusted)	0	0.01	0.20
95% CI	Referent	−0.09, 0.11	0.08, 0.31
β (Multivariate-adjusted)	0	−0.002	0.19
95% CI	Referent	−0.10, 0.09	0.08, 0.30

Abbreviations: FPG, fasting plasma glucose; HbA_1c_, hemoglobin A_1c_; β, partial regression coefficients.

The FPG and HbA_1c_ values are expressed as the means (SE).

Multivariate-adjustment was made for age, duration of diabetes mellitus, total energy intake, current drinking habits, regular exercise habits, depressive symptoms, oral hypoglycemic agents use, insulin use, and body mass index.

Table 3 indicates the adjusted mean values and partial regression coefficients (95% CIs) of FPG and HbA_1c_ according to the amount of smoking. Values of FPG did not increase in association with the amount of smoking. Conversely, HbA_1c_ increased significantly with increases in both number of cigarettes per day and pack-years of cigarette smoking compared with never smokers, indicating a dose-response relationship (P for trend = 0.001 and <0.001, respectively). Multivariate adjustment for the confounding factors listed above did not attenuate the association. The mean increase in HbA_1c_ in current smokers with ≥30 cigarettes per day relative to that in never smokers was 0.21%. The corresponding increase in current smokers with ≥50 pack-years was 0.25%.

**Table 3:** Age-adjusted mean values and multivariate-adjusted partial regression coefficients (95% CIs) of FPG and HbA_1c_ according to the amount of smoking.

	Never smoker	Current smoker (cigarettes per day)			
		<20	20–29	≥30	*P* for trend
	(n = 505)	(n = 249)	(n = 254)	(n = 176)	
FPG (mmol/l) (Age-adjusted)	7.82 (0.10)	7.88 (0.14)	7.81 (0.14)	8.08 (0.17)	0.34
β (Multivariate-adjusted)	0	−0.03	−0.11	0.12	0.86
95% CI	Referent	−0.37, 0.31	−0.46, 0.23	−0.27, 0.51	
HbA_1c_ (%) (Age-adjusted)	7.30 (0.05)	7.43 (0.07)	7.50 (0.07)	7.57 (0.08)	0.001
β (Multivariate-adjusted)	0	0.15	0.18	0.21	0.005
95% CI	Referent	−0.002, 0.30	0.03, 0.34	0.04, 0.39	
	Never smoker	Current smoker (pack-years)			
		<30	30−49	≥50	*P* for trend
	(n = 505)	(n = 211)	(n = 270)	(n = 198)	
FPG (mmol/l) (Age-adjusted)	7.83 (0.10)	7.78 (0.16)	7.85 (0.14)	8.10 (0.16)	0.21
β (Multivariate-adjusted)	0	−0.13	−0.06	0.12	0.66
95% CI	Referent	−0.49, 0.24	−0.39, 0.28	−0.25, 0.49	
HbA_1c_ (%) (Age-adjusted)	7.31 (0.05)	7.41 (0.07)	7.46 (0.06)	7.62 (0.07)	<0.001
β (Multivariate-adjusted)	0	0.10	0.18	0.25	0.001
95% CI	Referent	−0.06, 0.27	0.03, 0.33	0.09, 0.42	

Abbreviations: FPG, fasting plasma glucose; HbA_1c_, hemoglobin A_1c_; β, partial regression coefficients.

The FPG and HbA_1c_ values are expressed as the means (SE).

Multivariate-adjustment was made for age, duration of diabetes mellitus, total energy intake, current drinking habits, regular exercise habits, depressive symptoms, oral hypoglycemic agents use, insulin use, and body mass index.

We conducted additional analysis to evaluate the association between duration since quitting cigarette smoking and the glycemic level (Table 4). BMI was more elevated in past smokers compared with current smokers, whereas HbA_1c_ decreased linearly with the years after smoking cessation (P for trend <0.001). A similar pattern was also found in FPG, although it did not reach significance. The graded relationships of HbA_1c_ remained significant after adjusting for the above-mentioned confounders, including BMI.

**Table 4:** Age-adjusted mean values of BMI, FPG and HbA_1c_ and multivariate-adjusted partial regression coefficients (95% CIs) of FPG and HbA_1c_ according to the years since quitting.

	Current smoker	Years since quitting in past smokers			
		<10	10–19	≥20	*P* for trend
	(n = 679)	(n = 485)	(n = 310)	(n = 511)	
BMI (kg/m^2^) (Age-adjusted)	23.4 (0.1)	23.9 (0.1)	24.1 (0.2)	23.6 (0.1)	0.19
FPG (mmol/l) (Age-adjusted)	7.85 (0.08)	7.72 (0.09)	7.72 (0.12)	7.65 (0.10)	0.12
β (Multivariate-adjusted)	0	−0.12	−0.14	−0.17	0.19
95% CI	Referent	−0.36, 0.12	−0.42, 0.14	−0.41, 0.08	
HbA_1c_ (%) (Age-adjusted)	7.47 (0.04)	7.30 (0.05)	7.34 (0.06)	7.23 (0.05)	<0.001
β (Multivariate-adjusted)	0	−0.17	−0.17	−0.23	<0.001
95% CI	Referent	−0.28, −0.06	−0.30, −0.04	−0.35, −0.12	

Abbreviations: BMI, body mass index; FPG, fasting plasma glucose; HbA_1c_, hemoglobin A_1c_; β, partial regression coefficients.

The BMI, FPG and HbA_1c_ values are expressed as the means (SE).

Multivariate-adjustment was made for age, duration of diabetes mellitus, total energy intake, current drinking habits, regular exercise habits, depressive symptoms, oral hypoglycemic agents use, insulin use, and BMI.

Finally, we examined the relationships of smoking status with HOMA2-IR, HOMA2-%B, HS-CRP, and adiponectin in the same manner as in Tables 3 and 4 (Table 5). HOMA2-IR and HS-CRP increased, adiponectin decreased gradually in association with increases in both number of cigarettes per day and pack-years of cigarette smoking in comparison with never smokers. However, HOMA2-%B did not show a dose-response relationship. With respect to smoking cessation, compared with current smokers, HOMA2-IR and HS-CRP decreased in parallel as the years since quitting increased. However, there was no tendency for HOMA2-%B or adiponectin. Multivariate-adjustment did not materially alter these relationships.

**Table 5:** Age-adjusted mean values of HOMA2-IR, HOMA2-%B, HS-CRP, and adiponectin according to the amount of smoking and years since quitting.

	Never smoker	Current smoker (cigarettes per day)			
		<20	20–29	≥30	*P* for trend
HOMA2-IR	1.01 (0.97, 1.05)	1.08 (1.02, 1.14)	1.11 (1.05, 1.17)	1.10 (1.03, 1.18)	0.008
HOMA2-%B	39.4 (37.6, 41.3)	40.1 (37.6, 42.8)	42.1 (39.4, 44.9)	39.7 (36.6 43.1)	0.42
HS-CRP (mg/l)	0.39 (0.34, 0.44)	0.51 (0.43, 0.61)	0.51 (0.43, 0.61)	0.57 (0.46, 0.71)	<0.001
Adiponectin (μg /ml)	8.30 (7.91, 8.71)	8.15 (7.61, 8.72)	7.25 (6.77, 7.76)	7.19 (6.62, 7.80)	<0.001
	Never smoker	Current smoker (pack-years)			
		<30	30–49	≥50	*P* for trend
HOMA2-IR	1.01 (0.97, 1.05)	1.06 (1.00, 1.13)	1.09 (1.03, 1.15)	1.13 (1.06, 1.21)	0.002
HOMA2-%B	39.4 (37.6, 41.3)	40.5 (37.7, 43.5)	41.8 (39.2, 44.6)	39.5 (36.7, 42.6)	0.53
HS-CRP (mg/l)	0.39 (0.34, 0.44)	0.49 (0.41, 0.59)	0.52 (0.44, 0.62)	0.57 (0.47, 0.70)	<0.001
Adiponectin (μg /ml)	8.28 (7.88, 8.69)	8.20 (7.61, 8.84)	7.31 (6.85, 7.81)	7.27 (6.73, 7.84)	<0.001
	Current smoker	Years since quitting in past smokers			
		<10	10–19	≥20	*P* for trend
HOMA2-IR	1.09 (1.05, 1.13)	1.08 (1.04, 1.13)	1.04 (0.99, 1.10)	0.98 (0.95, 1.02)	<0.001
HOMA2-%B	41.2 (39.5, 42.9)	41.6 (39.7, 43.5)	42.2 (39.8, 44.7)	40.6 (38.8, 42.6)	0.77
HS-CRP (mg/l)	0.55 (0.49, 0.61)	0.57 (0.51, 0.65)	0.48 (0.41, 0.56)	0.44 (0.39, 0.50)	0.005
Adiponectin (μg /ml)	7.75 (7.43, 8.09)	8.19 (7.80, 8.61)	8.05 (7.56, 8.56)	7.91 (7.52, 8.31)	0.61

Abbreviations: HS-CRP, high sensitivity C-reactive protein; HOMA2-%B, homeostasis model assessment of β-cell function; HOMA2-IR, homeostasis model assessment of insulin resistance.

HOMA2-IR, HOMA2-%B, HS-CRP, and adiponectin levels are presented as geometric means (95% CI).

## Discussion

In the present study, we showed that active smoking was dose-dependently associated with increased levels of HbA_1c_ compared with never smoking habits in Japanese male patients with type 2 diabetes mellitus. Moreover, HbA_1c_ levels decreased in former smokers as the years since quitting smoking increased, in comparison with current smokers. These relationships remained significant even after adjusting for confounding factors, namely, age, duration of diabetes mellitus, total energy intake, current drinking habits, regular exercise habits, depressive symptoms, oral hypoglycemic agent use, insulin use, and BMI. Additionally, similar tendencies were also observed in relation to insulin resistance, HS-CRP, and adiponectin levels, which may explain its underlying pathological mechanisms. To our knowledge, this is the first large-scale epidemiological study to show a graded relationships of the amount of cigarettes smoked and years since quitting with the glycemic control in patients with type 2 diabetes mellitus.

To date, a number of epidemiological studies have indicated the association between cigarette smoking and elevated risk of type 2 diabetes mellitus. A meta-analysis of cohort studies [6] demonstrated that active smokers had an increased risk of type 2 diabetes mellitus (a pooled adjusted relative risk of 1.44 [95% CI 1.31, 1.58]) compared with never smokers. Furthermore, this association was stronger for heavy smokers (≥20 cigarettes/day; relative risk 1.61 [95% CI 1.43, 1.80]) compared with lighter smokers (<20 cigarettes/day; relative risk 1.29 [95% CI 1.13, 1.48]), indicating a dose-response relationship. Former smokers also had a higher risk of type 2 diabetes mellitus (relative risk of 1.23 [95% CI 1.14, 1.33]) [6]. Recently, the Nurses’ Health Study demonstrated the elevated risk of type 2 diabetes mellitus among former smokers though that decreased as the years since quitting increased [7]. However, the extent to which smoking habits deteriorate the glycemic control of diabetic patients was not fully examined and the results were inconsistent. Some studies found that active smoking was related to higher HbA_1c_ levels [8,9], whereas others did not [10,11]. In the present study, active smoking was dose-dependently associated with higher HbA_1c_ levels, supporting the hypothesis of positive relationship. Additionally, the present study showed a graded inverse association between the years since quitting and HbA_1c_, suggesting the reversible deleterious effect of smoking on glycemic control. Taken together, these findings may strengthen the benefit of smoking cessation for diabetic patients.

There are several plausible pathophysiologic mechanisms to explain the effect of smoking on glycemic control. Reportedly, cigarette smoking is associated with insulin resistance [10,11,18]. Smokers were shown to have elevated levels of systemic inflammation [19], oxidative stress biomarkers [20], and sympathetic activity [21]. Increases in cortisol and growth hormone were induced by nicotine from smoking [22]. These factors may contribute to insulin resistance. Further, higher levels of waist circumference in spite of lower BMI in current smokers [23], probably mediated by the anti-estrogenic effect of smoking [24,25], might play a part in increasing insulin resistance. Another possible pathway mediating this relationship may be a decrease in β cell function [26] as a consequence of chronic pancreatic inflammation [27] and pancreatic fibrosis [28]. On the basis of these reports, the adverse effects of cigarette smoking on glycemia seem to be mediated through both insulin resistance and impaired insulin secretion. In this study, HOMA2-IR levels showed a dose-response relation with the increasing amount of smoking (Table 5). Estimates of insulin resistance derived from HOMA is an indirect parameter compared with glucose clamp tests, a golden standard. However, it may be appropriate for use in large epidemiological studies [29], attributable to its simple and convenient nature. This use in diabetic patients showed a strong correlation with the insulin resistance index assessed by euglycemic–hyperinsulinemic clamp (r = −0.725, P <0.0001) [30]. Another study also showed a strong correlation between clamp-measured total glucose disposal and HOMA-estimated insulin sensitivity (r = −0.820, P <0.0001) in subjects including diabetic patients, with no substantial difference between nondiabetic (r = −0.754) and diabetic patients (r = −0.695) [31]. Though the cautious interpretation is needed, the use of HOMA model in subjects treated by insulin or insulin secretagogues may be applicable [29]. Some prior studies reported the relationship between smoking and insulin resistance, but the results were inconsistent [10,11,18,26]. In an experimental study among 40 healthy volunteers [18], smokers presented higher plasma insulin concentrations in response to oral glucose load despite similar glucose concentrations, and higher steady-state plasma glucose concentration compared with never smokers. The smoking-insulin resistance association was also observed in diabetic patients in earlier studies, though the numbers of subjects were relatively small (40 [10] and 52 patients [11], respectively). In contrast, another community-based cross-sectional study in Sweden showed no association between smoking status and insulin resistance [26]. The dose-response relationship of our study supports the hypothesis of positive association in a large number of patients. Further, the current study showed this association in past smokers, with monotonic decreases in HOMA2-IR with an increasing years since quitting (Table 5), which implies the reversible effect of smoking on insulin resistance. Furthermore, similar tendencies were observed for HS-CRP and adiponectin, which suggest the possible role of a low-grade inflammatory state and adipocytokine in insulin resistance. To the contrary, the association with HOMA2-%B was not observed, although male smoker had a lower HOMA β cell value in a prior community-based study [26]. The reason for the inconsistency is not clear, but it is possible that the detrimental effect of smoking on β cell function might be less apparent in diabetic patients with impaired insulin secretion. Moreover, a decreased insulin secretion capacity has been reported in Japanese individuals compared with Caucasian individuals [32], which suggests the existence of racial differences and may also explain the discrepancy.

In comparison to HbA_1c_, the association of smoking with FPG was weaker. Similar results were observed in a few earlier studies. A study among 96 patients with type 2 diabetes mellitus revealed that HbA_1c_ level was elevated significantly in smokers compared with never smokers, but fasting glycemia was not [8]. In a recent meta-analysis of nondiabetic subjects, Soulimane et al. [33] showed the higher HbA_1c_, lower 2-h plasma glucose in current smokers compared with never smokers, whereas no significant difference was observed for FPG. These findings may imply that smoking increases HbA_1c_ levels by increasing early postprandial plasma glucose levels, mediated through accelerated gastric emptying in smokers [34,35]. Regarding this discrepant results of glycemic parameters, Soulimane et al. [33] also mentioned the possibility of effect of increased passage of glucose across the erythrocyte membrane into the cell [36], rapid glycation of deoxyhemoglobin [37], increased rate of HbA_1c_ formation with elevated 2,3-diphosphorglycerate concentrations [37], and increased erythrocyte lifespan by carbon monoxide [38]. However, in the present study, the similar positive tendencies were also observed for HOMA2-IR (Table 5), which suggests the independent adverse effect of smoking on glycemic control.

Strengths of the current study are enrolment of a relatively large number of patients with type 2 diabetes mellitus, and the ethnicity of the study population. Considering the epidemic of diabetes mellitus in the Asian population, being characterized by impaired insulin secretion [32] and high smoking rate [4], these strengths may broaden the generalizability of the results. Additional strength is the use of confounding factors, such as total energy intake and depressive symptoms. Although they are closely associated with glycemic control, they were not always used as covariates in previous studies. Further, we showed the graded relationship of glycemic level with the amount of smoking and the inverse graded association with years since quitting, and both of these findings may strengthen the merit of smoking cessation.

Some limitations of our study should be discussed. First, we did not assess the presence of passive smoking habit, which has been associated with elevated risk of diabetes mellitus [7]. Therefore, the possibility of confounding by passive smoking cannot be denied. However, exposure to passive smoke in never smokers would likely bias our results toward the null hypothesis of no association. Therefore, the true association may be stronger than that shown in our study. Second, the present study included only male participants owing to the very low prevalence of female smokers (27% in male vs 7% in female) attributed to the Japanese cultural background. Though it may be due to the small number of female smoker, there is no statistically significant difference in HbA_1c_ levels among the smoking status. The age-adjusted mean values of HbA_1c_ were 7.54± 0.03 (SE) % for never smoker (n = 1627), 7.57 ± 0.09% for past smoker (n = 156), and 7.55 ± 0.10% for current smoker (n = 125) (P = 0.97). Therefore, the applicability of the present findings to female diabetic patients remains to be elucidated. Third, the cross-sectional design of our study does not allow for inferring any cause-and-effect relationships. Finally, there may be other confounding factors besides those evaluated in the present study.

In conclusion, the findings of the current study clearly showed the dose-response relationship of active smoking and smoking cessation with glycemic control in Japanese male patients with type 2 diabetes mellitus. Furthermore, similar associations were also observed for insulin resistance and systemic micro-inflammation. Therefore, active smokers may need to be encouraged quitting cigarette smoking for the clinical management of type 2 diabetes mellitus.
